# Optical Imaging of Tumor Response to Hyperbaric Oxygen Treatment and Irradiation in an Orthotopic Mouse Model of Head and Neck Squamous Cell Carcinoma

**DOI:** 10.1007/s11307-015-0834-8

**Published:** 2015-02-28

**Authors:** Joanna A. M. Braks, Linda Spiegelberg, Senada Koljenovic, Yanto Ridwan, Stijn Keereweer, Roland Kanaar, Eppo B. Wolvius, Jeroen Essers

**Affiliations:** 1 Department of Oral and Maxillofacial Surgery, Erasmus Medical Center, PO Box 2040, 3000 CA Rotterdam, The Netherlands; 2 Department of Pathology, Erasmus Medical Center, PO Box 2040, 3000 CA Rotterdam, The Netherlands; 3 Department of Genetics, Erasmus Medical Center, PO Box 2040, 3000 CA Rotterdam, The Netherlands; 4 Department of Otorhinolaryngology and Head & Neck Surgery, Erasmus Medical Center, PO Box 1738, 3015 CE Rotterdam, Netherlands; 5 Department of Radiation Oncology, Erasmus Medical Center, PO Box 2040, 3000 CA Rotterdam, The Netherlands; 6 Department of Vascular Surgery, Erasmus Medical Center, PO Box 2040, 3000 CA Rotterdam, The Netherlands

**Keywords:** Hyperbaric oxygen therapy, Radiation, Optical imaging, Near infrared fluorescence, Squamous cell carcinoma, Lymph node metastasis, Animal model, Head and neck cancer

## Abstract

**Purpose:**

Hyperbaric oxygen therapy (HBOT) is used in the treatment of radiation-induced tissue injury but its effect on (residual) tumor tissue is indistinct and therefore investigated in this study.

**Procedures:**

Orthotopic FaDu tumors were established in mice, and the response of the (irradiated) tumors to HBOT was monitored by bioluminescence imaging. Near infrared fluorescence imaging using AngioSense750 and Hypoxisense680 was applied to detect tumor vascular permeability and hypoxia.

**Results:**

HBOT treatment resulted in accelerated growth of non-irradiated tumors, but mouse survival was improved. Tumor vascular leakiness and hypoxia were enhanced after HBOT, whereas histological characteristics, epithelial-to-mesenchymal transition markers, and metastatic incidence were not influenced.

**Conclusions:**

Squamous cell carcinoma responds to HBOT with respect to tumor growth, vascular permeability, and hypoxia, which may have implications for its use in cancer patients. The ability to longitudinally analyze tumor characteristics highlights the versatility and potential of optical imaging methods in oncological research.

**Electronic supplementary material:**

The online version of this article (doi:10.1007/s11307-015-0834-8) contains supplementary material, which is available to authorized users.

## Introduction

Head and neck squamous cell carcinoma (HNSCC) is the sixth most common cancer type worldwide and is associated with a poor prognosis. Treatment of these cancers often involves surgical resection followed by radiotherapy. Despite advances in radiation protocols that minimize the targeted tissue volume, radiation treatment often causes considerable damage to the surrounding healthy tissues resulting in complications like impaired wound healing and osteoradionecrosis. Hyperbaric oxygen therapy (HBOT) is frequently used in the management of radiation-induced tissue injury, and has shown beneficial effects although its working mechanism has not completely been unraveled yet [1, 2]. In HBOT, patients inspire 100 % oxygen at elevated barometric pressure, which enhances the amount of oxygen that is dissolved in the plasma leading to an increase of the oxygen tension in tissues. By creating an oxygen gradient, HBOT is thought to induce neovascularization by which the progressive loss of the microvasculature in hypoxic irradiated tissue may be overcome and tissue healing is improved [3–5].

The use of HBOT in patients with a history of cancer has often raised concerns about the promoting effect this therapy might have on the growth of (residual) tumor tissue. Poor oxygenation and abnormal vasculature is a common feature of solid tumors and reduces the ability of cells to divide. It was anticipated that by its pro-angiogenic effect, HBOT would stimulate cancer growth and recurrence [6–8]. On the other hand, tumor hypoxia is known to be essential for the progression of cancer and is related to increased cell survival, induction of angiogenesis, metastasis, and therapy resistance [9, 10]. Enhanced oxygenation of tumors by HBOT could therefore lead to less aggressive cancer growth and a better prognosis. Based on clinical and experimental studies, it was recently adopted that there is no evidence that HBOT has a cancer-promoting effect [11, 12]. Even more, on certain cancer subtypes like gliomas and mammary tumors, an anti-angiogenic and growth-inhibitory effect of HBOT was reported [13–15].

In most studies in which subcutaneously implanted squamous carcinoma cell lines in mice were used, no differences in growth between control and HBOT groups were seen [16–20]. However, in a recent study, Paniello *et al.* [21] reported enhanced growth of HNSCC tumor cells in C3H mice after HBOT. These divergent outcomes suggest that the choice of the experimental model, regarding cancer cell type, tumor location, or HBOT protocol, is critical for the proper determination of tumor responses to HBOT.

One of the tumor conditions that is relevant to the clinical situation but has been scarcely investigated in experimental HBOT studies is the irradiated tumor. Since radiation not only modifies the cancer cells but also the microenvironment of the tumor by affecting angiogenesis and the hypoxic state of the tissue [22], previous irradiation might well influence the response of the residual tumor to HBOT.

By using an improved, clinically relevant tumor model and advanced optical imaging strategies, we intend to increase the insight on the effects of hyperbaric oxygen on tumor growth and factors that influence tumor behavior like tumor vascularization, hypoxia, and differentiation. In the present study, we used bioluminescent imaging (BLI) to non-invasively and adequately monitor the growth of a human squamous cell carcinoma line (FaDu) in the floor of the mouth of immunodeficient mice. Near infrared fluorescence (NIRF) optical imaging was applied to detect and quantify the effects of HBOT and irradiation on specific tumor characteristics *in vivo*. The fluorescent blood pool agent AngioSense was used to analyze tumor blood vessel quality, and the NIRF targeting probe HypoxiSense was applied to study hypoxia in the tumors. Furthermore, this orthotopic mouse model allowed us to investigate the effects of HBOT on the development of regional and distant metastases, which are likewise frequently seen in patients with HNSCC.

## Materials and Methods

### Mice

All animal experiments of this study were approved by the Animal Experiments Committee of the Erasmus Medical Center (DEC 2645). The Dutch Experiments on Animal Act is established under the European guidelines (EU Directive No. 86/609/EEC regarding the Protection of Animals used for Experimental and Other Scientific Purposes). BALB/c nu/nu female mice (Charles River Laboratories), aged 8 to 11 weeks, were kept in filter-top cages with autoclaved pellet food and sterilized water without restriction. Mice with tumors in the floor of the mouth were given soft food and were monitored daily. Animals were euthanized when they had lost more than 20 % of their initial body weight or had reached day 35 after tumor implantation.

### Tumor Generation

The human hypopharyngeal squamous cell carcinoma line FaDu-luc2 was kindly received from the laboratory of Prof. C.W. Löwik, PhD (Leiden University Medical Center, Leiden, The Netherlands). This cell line had been transfected with a luciferase-expressing vector (pCAGGS- Luc-2) allowing the monitoring of the tumor growth by bioluminescence imaging (BLI) [23]. FaDu-luc2 cells were grown in Dulbecco’s modified Eagle’s medium (DMEM, Lonza) supplemented with 10 % (*v*/*v*) fetal bovine serum (Hyclone) and antibiotics (50 units/ml of penicillin and 50 μg/ml streptomycin) at 37 ° C in a humidified atmosphere of 5 % CO_2_ in air.

Orthotopic tumors were established by transcervical injection of 1 × 10^5^ cultured FaDu-luc2 cells, suspended in 20 μl serum-free DMEM into the floor of the mouth of anesthetized (2–4 % isoflurane) nude mice.

### Hyperbaric Oxygen Treatment (HBOT)

Treatment with hyperbaric oxygen started at day 5 after tumor implantation and consisted of daily sessions, until the end of the experiment with a maximum of 30 sessions. The hyperbaric oxygen chamber used in this study was custom-built for small laboratory animals (Hytech BV, Raamsdonksveer, The Netherlands) [24]. Each session started with a compression phase of 15 min, during which the pressure in the chamber was elevated to 2.4 atm absolute (ATA) and the oxygen level to 100 %. After 90 min of isopression, decompression to 1 ATA took place in 15 min.

### Radiation Therapy (RT)

Mice were anesthetized (65 mg/kg ketamine and 10 mg/ml xylazine) and locally irradiated at day 5 after tumor implantation with a single dose of 5 Gy using a Gammacell 40 Exactor Cs-137 γ-source. With this dose, tumor growth is significantly reduced, but not all tumor cells are killed. Mice were shielded using a Gammacell 40 Collimator centering the head and neck region in a 3-cm radiation field.

### Bioluminescence Imaging (BLI)

Tumor growth was monitored twice a week by bioluminescence imaging using an IVIS Spectrum Imaging System (Xenogen). An aqueous solution of luciferin (Caliper Life Sciences) at 150 mg/kg was injected intraperitoneal 10–20 min before imaging. Animals were anesthetized (2–4 % isoflurane) and placed in a dorsal position during imaging. Using the Living Image software 3.2 (Xenogen), photon flux was quantified within a circular region of interest (ROI) encompassing the head and neck region of each mouse. For 3D reconstruction, bioluminescence imaging (BLI) images were coregistered with computed tomography (CT) images.

### Fluorescence Molecular Tomography (FMT) Imaging

One day prior to their endpoint and at least 20 h after the last HBOT session, mice were intravenously injected with 1.3 nmol of the fluorescent blood pool imaging agent AngioSense750 (PerkinElmer) and/or 1.3 nmol of the carbonic anhydrase IX (CAIX) targeted fluorescent imaging agent HypoxiSense680 (PerkinElmer) or MMPSense680, a probe that is activated after cleavage by matrix metalloproteinases (MMPs). For quantitative fluorescence molecular tomography imaging (FMT 2500, PerkinElmer), mice were anesthetized (2–4 % isoflurane) and fixed in a definite position in an animal imaging cassette. The FMT 2500 tomography software was used to quantitate fluorochrome concentration distribution of AngioSense in a ROI of 750 mm^3^ in the tumor area. *In vivo* imaging sessions were performed 2 and 24 h post-injection and hereafter, mice were euthanized (isoflurane overdose) and the tumors were dissected for *ex vivo* imaging. For multi-modality imaging, image data from FMT were fused with CT using markers in the multimodal mouse bed.

### Histology

Mouse tumors were fixed in 10 % formalin, embedded in paraffin, and 5 μm slides were cut. Routine hematoxylin and eosin (H&E) staining was performed and assessed by a pathologist. For immunohistochemistry, slides were probed with primary antibodies against Ki67 (Novus Biologicals Ltd.) and CD31 (Abcam) to assess proliferation and blood vessel density and diameter, respectively. Biotinylated goat anti-rabbit IgG (Dako) was used as secondary antibody, and detection was performed with streptavidin–peroxidase (R&D Systems) and 3,3′-diaminobenzidine (Dako). Hematoxylin served as counterstain. Slides were scanned using a slide scanner (Hamamatsu Photonics). To measure proliferation, the percentage of Ki67-positive cells per tumor area was determined by using Cell^d^ (Olympus Life Science Europe GmbH). Apoptosis levels were determined by counting the number of apoptotic cells in proliferating tumor areas (×20) in H&E-stained slides. To determine vascular density, CD31-positive blood vessels were counted in 20 representative fields (×40) for each tumor. The vascular diameter of 30 vessels for each tumor was measured in ×63 high power fields.

### Metastasis

During the course of the experiment, the development of metastases was monitored by BLI of the total mouse body. Imaging was performed using unmixed emission spectra, allowing signal detection at particular tissue depths, which prevented outshining of the signal of the regional metastasis by the primary tumor. To establish the incidence of lymph node metastases, two superficial cervical lymph nodes were resected from each mouse, incubated for 10 min in luciferin solution (30 μg/ml), and *ex vivo* BLI was performed. To confirm the metastatic lesions, lymph nodes were embedded in paraffin, sectioned, and H&E-stained.

### Quantitative Real-Time Reverse Transcription Polymerase Chain Reaction (qPCR)

Mouse tumors were dissected, rapidly frozen in liquid nitrogen, and stored at −80 °C. Total RNA was isolated using the RNeasy Mini Kit (Qiagen) and reverse transcribed using the iScript cDNA Synthesis Kit (Bio-Rad). cDNA was amplified in 40 cycles (20 s 95 ° C, 3 s 95 ° C, 30 s 60 ° C) with a Bio-Rad cycler using Fast SYBR Green Master Mix (Applied Biosystems). Specific primers (see supplementary Table S1) were used to amplify cDNA of human vascular endothelial growth factor A (VEGF), carbonic anhydrase IX (CAIX), E-cadherin (CDH1), Vimentin (Vim), snail family zinc finger 1 (Snail), transforming growth factor beta 1 (TGFβ1), and the internal control glyceraldehyde-3-phosphate dehydrogenase (GAPDH). Each PCR reaction was performed in duplicate, and the average threshold cycle (Ct) value was used for relative quantification of gene expression with the comparative Ct method (ΔΔCT).

### Statistical Analysis

Data are expressed as mean values with standard error of the mean (SEM), and were analyzed using SPSS PASW 21.0 for Windows (SPSS Inc., Chicago, USA). The Shapiro-Wilk test was used to test for normality, followed by the Mann-Whitney *U* test for the comparison of non-normally distributed data, while Student’s *t* test was used for normally distributed data. *p* < 0.05 indicated significant differences. Survival data were analyzed by the Kaplan-Meier and log-rank tests for survival distribution. The Fisher’s exact test was used to analyze differences in the incidence of lymph node metastasis between groups.

## Results

### Effect of HBOT on Tumor Growth and Mouse Survival

**Fig. 1:**
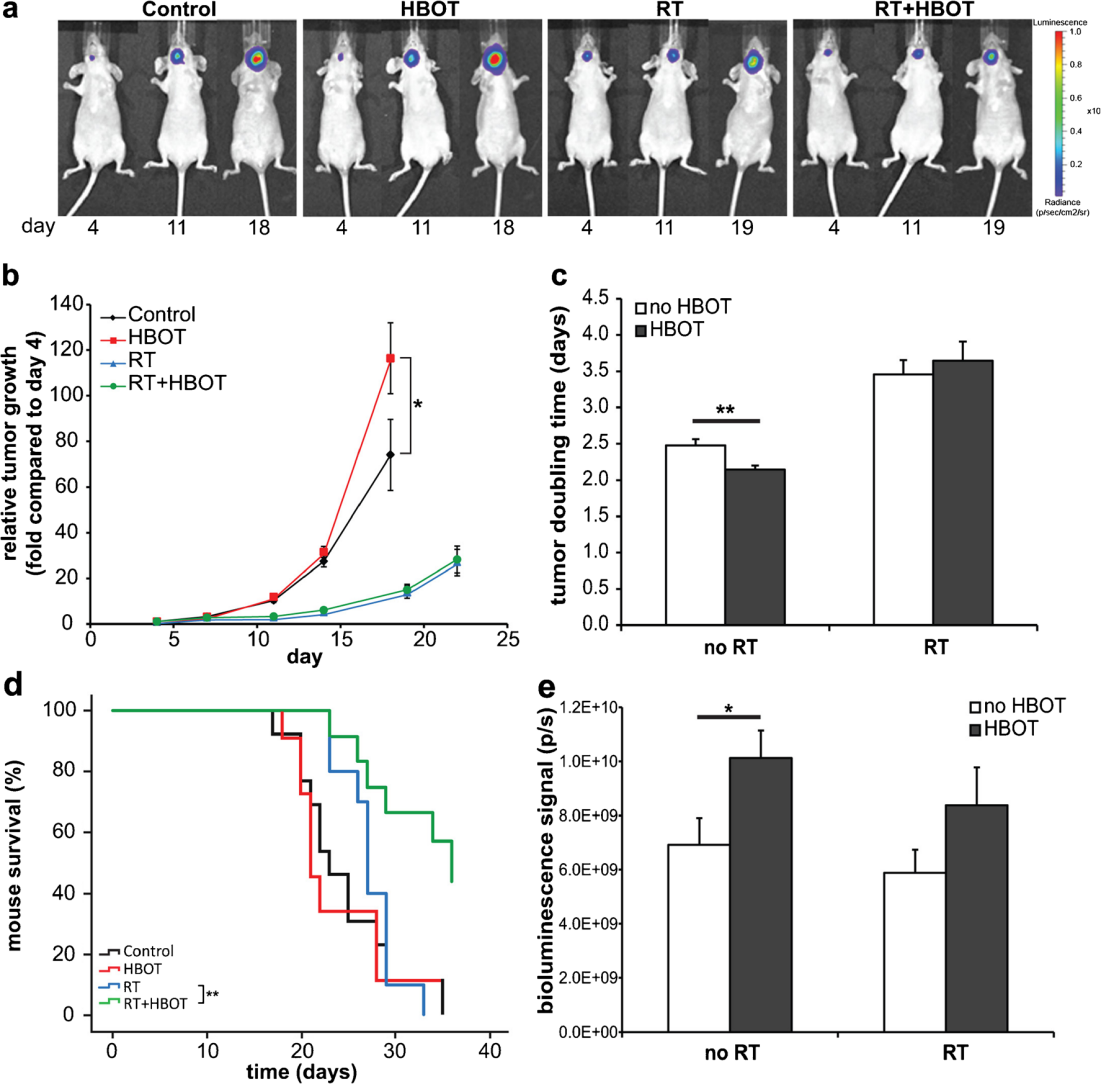
*In vivo* effects of HBOT on tumor growth and survival time in FaDu-luc tumor-bearing mice. **a** Bioluminescence imaging (*BLI*) of representative mice of the different treatment groups on day 4, 11, and 18 or 19 after xenografting tumor cells in the floor of the mouth. **b** Increase of BLI signals compared to day 4 after xenografting tumor cells (9–12 mice per group). For the fold change numbers, see supplementary Table S2. **c** Mean doubling times of the tumors based on the BLI signals measured between day 7 and 18 (no RT) or day 11–22 (RT). **d** Mouse survival time analysis using the Kaplan-Meier method and comparisons using log-rank tests. **e** Mean BLI signals of the tumors at the endpoint. *Error bars* indicate SEM. ** *p* < 0.01; *p < 0.05. *RT* radiation therapy, *HBOT* hyperbaric oxygen therapy.

FaDu tumors were implanted in the floor of the mouth of nude mice, and the growth was monitored by bioluminescence imaging (BLI) until they met criteria for euthanasia, mostly due to weight loss. As shown in Fig. 1, the increase of the BLI signal was significantly higher (*p* = 0.023) in the group of mice that had undergone daily treatments of HBOT compared with the untreated group on day 18 after tumor cell inoculation (Fig. 1a, b). The mean doubling times for the BLI signals of the individual tumors were significantly lower in the HBOT group *versus* the control (2.15 vs 2.47 days, *p* = 0.006) (Fig. 1c). In irradiated tumors, the tumor growth rate was delayed compared to non-irradiated tumors, but no significant effect of HBOT was observed here (doubling times 3.46 and 3.65 days) (Fig. 1b, c).

The median survival periods for mice in the control, HBOT, RT, and RT + HBOT groups were 23, 21, 27, and 36 days, respectively. There was no significant effect of HBOT on the survival of non-irradiated mice, but mice with irradiated tumors had an increased survival time if HBOT had been applied (*p* = 0.003) (Fig. 1d). The maximal BLI values measured at the time of euthanasia were higher in the HBOT group as compared to the control for both non-irradiated (*p* = 0.020) and irradiated tumors (not significant, *p* = 0.176) (Fig. 1e).

### Effect of HBOT on Tumor Vascularization and Vascular Permeability

**Fig. 2:**
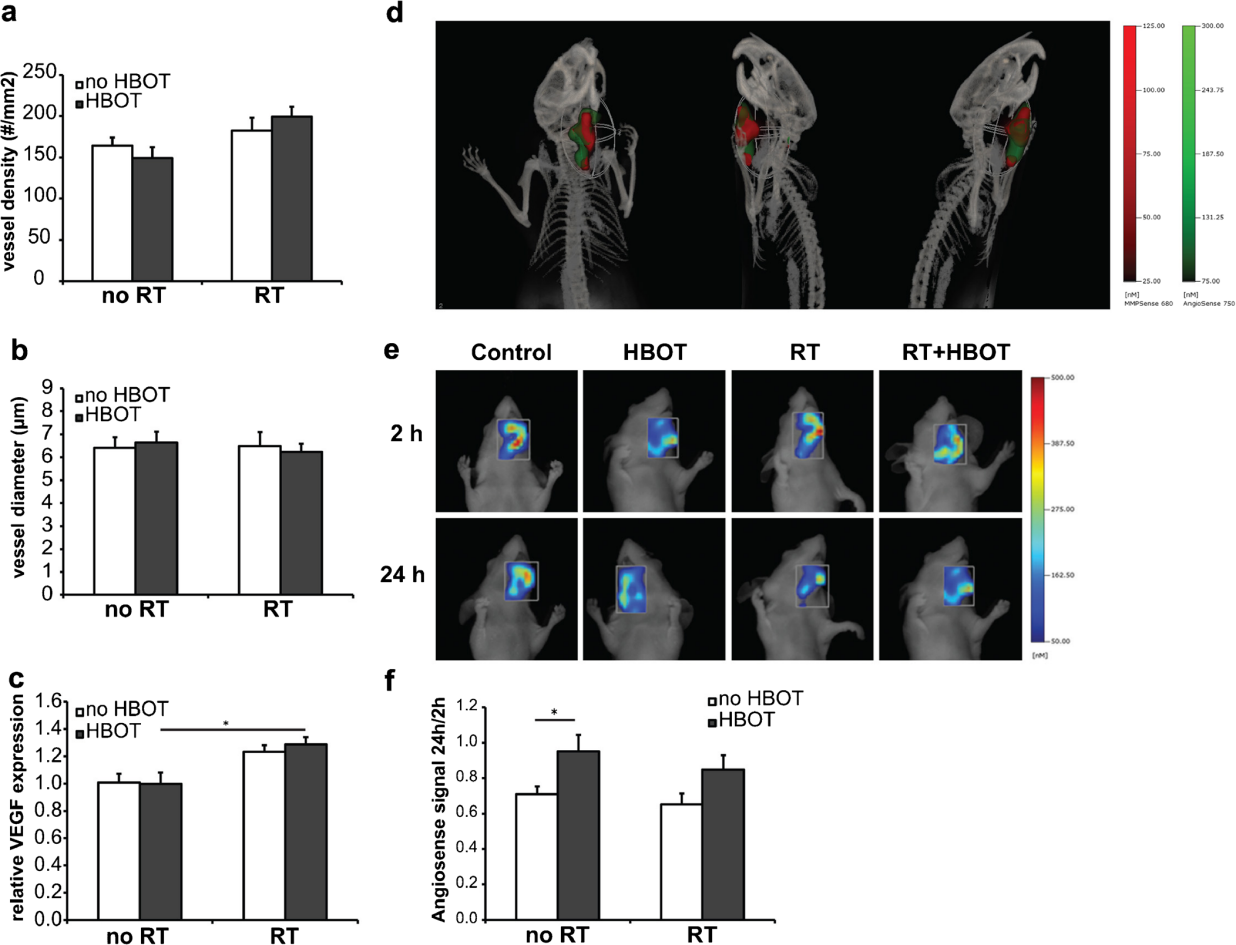
Effect of HBOT on tumor vasculature. **a** Quantification of the tumor blood vessel density. **b** Quantification of the tumor blood vessel diameter. **c** Relative expression levels of VEGF mRNA in the tumors. **d** Multimodal FMT/CT imaging of a FaDu-luc mouse 24 h after injection of MMPSense680 (*red*) and AngioSense750 (*green*) to detect tumor margins and region of tumor vascular leak, respectively. **e** Representative FMT images of tumor regions in FaDu-luc mice of the different treatment groups, 2 and 24 h after AngioSense750 injection. **f** Quantification of blood vessel leakage in the tumor regions. For each animal, the 24 h/2 h AngioSense signal ratio was determined (*n* = 6). *Error bars* indicate SEM. *p < 0.05. *RT* radiation therapy, *HBOT* hyperbaric oxygen therapy.

Vascularization of the tumors was analyzed by investigating the CD31-positive blood vessels in tumor sections. The mean blood vessel density was slightly increased in irradiated tumors (1.2-fold, *p* = 0.014) but no significant effect of HBOT was observed (Fig. 2a). The mean tumor blood vessel diameter did not differ between the groups (Fig. 2b). mRNA levels of VEGF, a key factor involved in angiogenesis, were significantly increased in the irradiated tumors (1.3-fold, *p* = 0.000), but not affected by HBOT (Fig. 2c).

Tumor blood vessel quality was analyzed *in vivo* with FMT using AngioSense750 as a blood pool marker. AngioSense remains in the vasculature for 0–4 h, and therefore the signal detected in the tumor area 2 h after probe injection is a measure for the tumor vascular volume. The degree of AngioSense retention in the tumor area after 24 h is indicative for vascular leakiness [25, 26]. In Fig. 2d, the site of accumulation of AngioSense750 in and around the tumor is shown and coregistered with the signal of the probe MMPSense680 which indicates the tumor margins. For each individual mouse, the ratio between the 24 h and 2 h AngioSense signal was determined and probe accumulation appeared to be higher in the mice treated with HBOT in both the non-irradiated (1.3-fold, *p* = 0.042) and the irradiated group (1.3-fold, *p* = 0.078), indicating an increase in tumor vascular permeability after HBOT [Fig. 2e, f].

### Effect of HBOT on Tumor Hypoxia

**Fig. 3:**
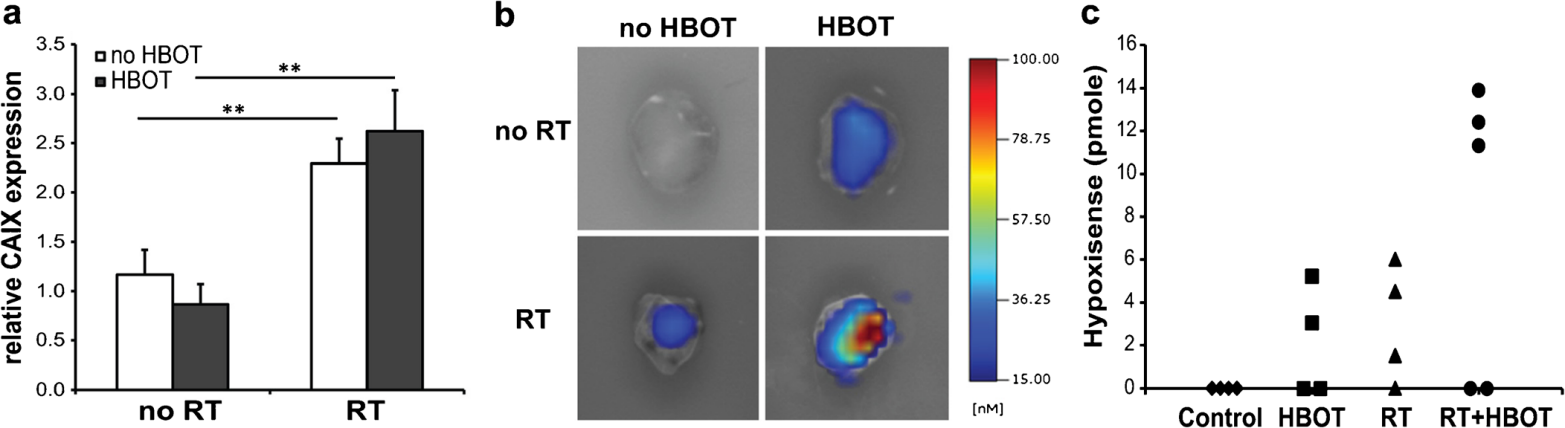
Effect of HBOT on tumor hypoxia. **a** Relative expression levels of CAIX mRNA in the tumors as determined by qPCR. *Error bars* indicate SEM. **p < 0.01. **b** Representative *ex vivo* FMT images of dissected FaDu-luc tumors 24 h after HypoxiSense680 injection. **c** Quantification of HypoxiSense signals in individual dissected tumors. *RT* radiation therapy, *HBOT* hyperbaric oxygen therapy.

In tumors of irradiated animals, a clear increase in mRNA levels of the hypoxia inducible factor CAIX (2.4-fold, *p* = 0.000) was observed, but no significant effect of HBOT on CAIX expression was detected (Fig. 3a).

Tumor hypoxia was further analyzed by FMT using the CAIX targeted fluorescent imaging agent HypoxiSense680 as a probe. Because of the low fluorescence levels, it was not possible to obtain *in vivo* data regarding the hypoxic state of the tumors. Mice were sacrificed 24 h after probe injection and analyzed *ex vivo* (Fig. 3b). HypoxiSense signals were detected in none of the control tumors but half of the HBOT tumors. Stronger HypoxiSense signals were observed in the tumors of the irradiated animals with again the highest fluorescent levels in the HBOT group (Fig. 3b, c), indicating that tumor hypoxia is increased after HBOT.

### Effect of HBOT on Tumor Pathological Features and Metastasis

**Fig. 4:**
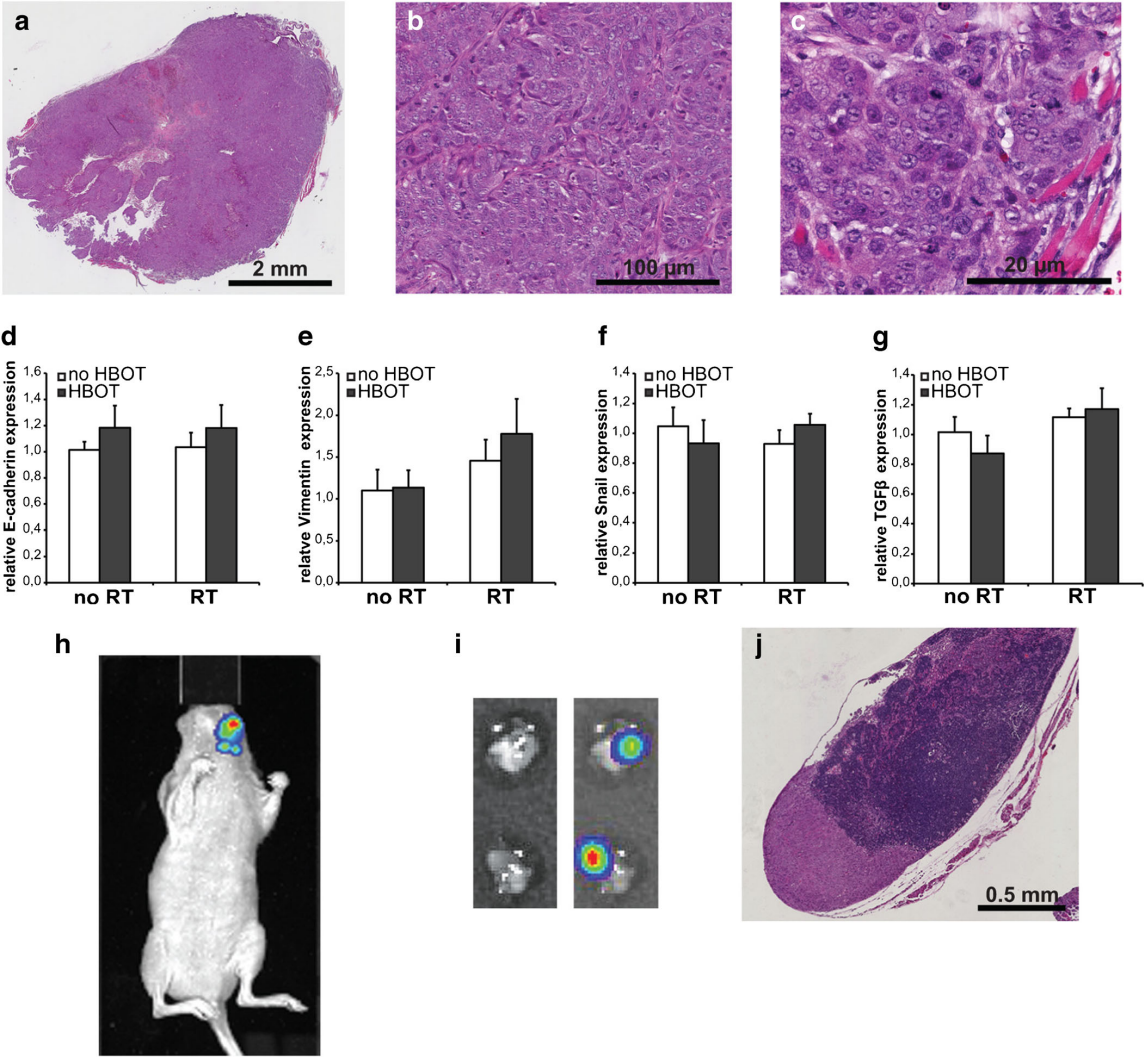
Effect of HBOT on tumor malignancy parameters. **a** H&E staining of a tissue section of a representative FaDu-luc tumor dissected from the floor of the mouth. All treatment groups show poorly to moderately differentiated squamous cell carcinoma. **b** Detail of A. **c** Detail of A. **d**–**g** Relative expression levels of E-cadherin (**d**), Vimentin (**e**), Snail (**f**), and TGFβ1 (**g**) mRNA in the tumors. *RT* radiation therapy, *HBOT* hyperbaric oxygen therapy. **h** *In vivo* BLI of a tumor-bearing mouse at day 20 at an emission wavelength of 560 nm showing lymph node metastases. **i** Photographic (*left*) and bioluminescent (*right*) images of metastasized cervical lymph nodes. **j** H&E staining of a tissue section of a representative metastasized lymph node.

The tumors were rated as poorly to moderately differentiated squamous cell carcinoma with moderate infiltrative borders for all experimental groups (Fig. 4a, b, c). Perineural growth and vascular invasion of tumor cells was evident in several tumor sections but no significant differences were observed between the HBOT and the control groups. The degree of necrosis (Table 1) was highly variable among the tumors (0–42.2 % of the tumor area) and was related to tumor size, but no significant effect of HBOT was established. Tumor cell proliferation and apoptosis levels were also not significantly affected by HBOT although a trend towards decreased cell death after HBOT was observed (Table 1).

**Table 1:** Proliferation, apoptosis, and necrosis in FaDu tumors

Factor	Control	HBOT	RT	RT + HBOT
Proliferation (% Ki67-positive tumor cells)^a^	38.1 ± 1.8	40.0 ± 1.5	43.2 ± 1.7	41.9 ± 1.2
Apoptosis (# cells/mm^2^)^a^	30.6 ± 2.9	24.9 ± 2.4	22.2 ± 3.9	19.0 ± 4.1
Necrosis (% area)^b^	2.9 [0–28.0]	6.9 [0.5–27.5]	5.0 [0–42.1]	6.9 [0–31.0]

*RT* radiation therapy, *HBOT* hyperbaric oxygen therapy

^a^Mean values ± SEM

^b^Median values [range]

To investigate the impact of HBOT on epithelial-to-mesenchymal transition (EMT), mRNA expression levels of the malignancy markers E-cadherin, Vimentin, Snail, and TGFβ1 were determined. Vimentin expression was slightly upregulated in the irradiated tumors (1.4-fold, *p* = 0.039) but its levels were not significantly influenced by HBOT (Fig. 4e). On the tumor expression of E-cadherin, Snail, and TGFβ1, no significant effects of either RT or HBOT were observed (Fig. 4d, f, g).

The orthotopic tumor model allowed us to identify and monitor the development of regional or distant metastases in real time by *in vivo* BLI using spectral unmixing and 3D reconstruction to circumvent outshining of the signals by the strong total BLI signals of the primary tumor in the floor of the mouth (Fig. 4h and Online video). In the time frame of the experiment, distant metastases were not detected but cervical lymph node metastases developed in the majority of the mice. To confirm and characterize the metastases, two superficial cervical lymph nodes of each mouse were harvested immediately after euthanasia and analyzed by *ex vivo* BLI and histology (Fig. 4i, j). For the non-irradiated as well as the irradiated mice, the metastatic incidence was not affected by HBOT (Table 2). Also, no effect of HBOT on the histological stage of the lymph node metastases was observed.

**Table 2:** Incidence and rate of lymph node metastasis in mice with FaDu tumors

Factor	Control	HBOT	RT	RT + HBOT
Metastatic incidence	8/12	8/11	9/10	10/11
Metastatic percentage	67 %	72 %	90 %	91 %
Fisher’s exact test vs control		1.000	0.323	0.317

*RT* radiation therapy, *HBOT* hyperbaric oxygen therapy

## Discussion

In this study, tumor responses to HBOT were investigated in an orthotopic mouse model of head and neck squamous cell carcinoma using optical imaging methods. By means of bioluminescence imaging, the growth of a human hypopharyngeal carcinoma cell line in the floor of the mouth of mice was accurately monitored and revealed a small but significant increase in tumor growth rate (19 %) under the influence of HBOT. No effect of HBOT, however, was detected on the growth of tumors that had been irradiated before. The difference in response might lie in the fact that irradiation, in addition to killing tumor cells, also damages endothelial cells [27, 28], resulting in a tumor microenvironment that is less susceptible to HBOT-induced, growth promoting stimuli. Although tumors grew faster in HBOT-treated mice as compared to controls, the survival time of these animals was not affected. Interestingly, the bioluminescent signals of the tumors at the endpoints were higher in the HBOT group, indicating that these mice survived higher loads of viable tumor tissue. Also, in the irradiated groups, in which the survival period was extended by HBOT but the tumor growth rate was not affected, a trend towards increased endpoint tumor load was noticed. HBOT might affect the disturbed metabolic processes in the body that lead to cancer-associated weight loss and in this way increase survival rates. From a clinical point of view, this is an interesting finding and future studies are warranted to investigate this potential beneficial impact of HBOT. Histological analyses could not reveal the factors that underlie the accelerated tumor growth after HBOT. Small and possibly transient influences of HBOT on vascularization, proliferation, or cell death might have lead to the observed growth effect. HBOT stimulates vessel development in normal tissue and in wounds [3, 5] but its effect on tumor vascularization is unclear. Tumors possess disorganized and leaky tumor vessels which block adequate tissue perfusion leading to the presence of hypoxic regions that are associated with poor prognosis and treatment outcome [29, 30]. Normalization of the tumor vasculature is thought to lead to less tumor hypoxia and is a goal of anti-angiogenic therapies [9, 31]. In our HNSCC tumor model, *in vivo* molecular imaging with the blood pool agent AngioSense disclosed a higher vascular leakiness in tumors of HBOT-treated animals. This is the first study exploring the effects of HBOT on vascular permeability, indicating that HBOT does not lead to normalization of blood vessels and might even deteriorate tumor vascular quality.

To compare the hypoxic states of the tumors, the recently developed HypoxiSense probe, which detects the protein CAIX on the tumor cell surface, was employed. Due to relatively low fluorescent signals, however, *in vivo* data could not be obtained. In previous studies, this fluorescent agent was successfully used in subcutaneous xenograft tumors with volumes of 600–700 mm [3, 32]. In our orthotopic model, the tumors in the floor of the mouth did not grow beyond 250 mm^3^ and therefore signal detection was probably hampered by optical properties such as background absorption and scattering [33]. Nevertheless, *ex vivo*, hypoxic regions were detected in a subset of tumors and the data suggest that irradiated tumors were more hypoxic than non-irradiated tumors and moreover, that HBOT aggravated tumor hypoxia as well. It has been demonstrated that HBOT increases the oxygen concentration in tumor tissue during and shortly after treatment but this effect is transient [34, 35]. The drop in oxygen level following a HBOT session may lead to the induction of a hypoxic response in the tumor tissue, by which CAIX expression could be enhanced. This would correspond to previous studies in which exposure to HBOT resulted in increased levels of the hypoxia inducible factor HIF-1ɑ in the liver and brain of rats [36–38]. It is also possible that CAIX production was not stimulated by hypoxia, but by inflammatory cytokines that are known for their ability to induce HIF-1ɑ stabilization as well [39]. The fact that CAIX mRNA levels were not found to be elevated in the dissected tumors suggests that CAIX gene activation had been transient, and that transcription had returned to normal levels since the last HBOT session. In conclusion, our data indicate that the use of intensive HBOT protocols does not lead to long-term overall reduction of tumor hypoxic responses.

The presence of cervical lymph node metastasis is an important prognostic indicator for patients with HNSCC. The current bioluminescent orthotopic tumor model allowed us to monitor the consequences of treatment for the development of lymph node metastases. Metastatic incidence was increased from approximately 70 to 90 % in the irradiated animals, but was not affected by HBOT. This confirms previous experimental results obtained in different cancer and animal models, in which stimulation of metastasis by HBOT was not established either [16, 40–44]. Histopathological and molecular analysis of the tumors did not reveal significant HBOT-induced changes in malignant parameters. Epithelial-to-mesenchymal transition (EMT) is a central mechanism of cancer metastasis whereby tumor cells are reprogrammed, resulting in decreased adhesion and enhanced migration and invasion [45–47]. The expression of the hallmark molecules of EMT, the epithelial marker E-cadherin, the mesenchymal marker Vimentin, and the EMT-inducing factors Snail and TGFβ1 was not affected by HBOT, indicating that there was no switch to more aggressive tumors. Moen *et al.* [15] reported induction of mesenchymal-to-epithelial transition (MET) by HBOT in a mammary tumor model, but thus far there are no indications for similar effects in squamous cell cancer.

Altogether, in this study, we found that HBOT stimulated the growth of non-irradiated tumors and increased tumor blood vessel leakiness and hypoxia. These are factors known to promote aggressive tumor behavior and poorer treatment outcome [10, 30]. On the other hand, HBOT was beneficial for animal survival and no effects of HBOT were detected on metastatic incidence, histological grade, and malignancy markers, suggesting that the effects of HBOT on disease outcome are limited and there might be no increased risk for negative effects of HBOT in patients that were previously subjected to radiation therapy. Previous experimental studies on the effects of HBOT on tumor behavior thus far yielded varying results [8, 11]. In mammary and glioma tumor models, growth-inhibiting and anti-angiogenic effects were reported using a HBOT schedule of 3 to 4 sessions per week [13, 14, 48]. Studies using squamous cell cancer models did not reveal effects of HBOT on tumor growth [16–20], except for a recent study by Paniello *et al.* [21] who also observed enhanced growth of xenografted HNSCC tumors in mice. In these studies, daily HBOT sessions were applied, according to the clinically used protocols for the management of radiation-induced injury. Therefore, specific tumor characteristics and HBOT time schedules might underlie the different outcomes and need to be considered in future studies.

## Conclusions

The improved animal model and *in vivo* molecular imaging methods used in this study disclosed influences of HBOT on the growth rate, blood vessel quality, and hypoxic state of squamous cell carcinoma and opens up possibilities to further investigate the circumstances and conditions in which HBOT can be safely used in cancer patients.

## Electronic Supplementary Material

Below is the link to the electronic supplementary material.

ESM 1 (PDF 95 kb)

3D multimodal imaging of a bioluminescent tumor in a mouse. A video representing 3D bioluminescence imaging coregistered with CT imaging of a mouse bearing a FaDu-luc-tumor in the floor of the mouth and detection of a lymph node metastatic lesion. (AVI 12.5 MB)
