# Supplementary material for: Optical Imaging of Tumor Response to Hyperbaric Oxygen Treatment and Irradiation in an Orthotopic Mouse Model of Head and Neck Squamous Cell Carcinoma
Source: Mol Imaging Biol. 2015 Feb 28;17(5):633–42. doi: 10.1007/s11307-015-0834-8 (PMC4768231; doi:10.1007/s11307-015-0834-8)
Supplement: Supplementary file 1 — (PDF 95 kb) [file 11307_2015_834_MOESM1_ESM.pdf]

Supplemental Information

Optical Imaging of Tumor Response to Hyperbaric Oxygen Treatment and Irradiation in an Orthotopic Mouse Model of Head and Neck Squamous Cell Carcinoma

Journal: Molecular Imaging and Biology

Joanna A.M. Braks[1], Linda Spiegelberg[1], Senada Koljenovic[2], Yanto Ridwan[3], Stijn Keereweer[4], Roland Kanaar[3,5], Eppo B. Wolvius[1], Jeroen Essers[3,5,6]

[1] Department of Oral and Maxillofacial Surgery, Erasmus Medical Center, PO Box 2040 3000 CA Rotterdam, the Netherlands.
[2]Department of Pathology, Erasmus Medical Center, PO Box 2040, 3000 CA Rotterdam, the Netherlands.
[3]Department of Genetics, Erasmus Medical Center, PO Box 2040, 3000 CA Rotterdam, the Netherlands.
[4]Department of Otorhinolaryngology and Head & Neck Surgery, Erasmus Medical Center, PO Box 1738, 3015 CE Rotterdam, Netherlands.
[5]Department of Radiation Oncology, Erasmus Medical Center, PO Box 2040, 3000 CA Rotterdam, the Netherlands.
[6]Department of Vascular Surgery, Erasmus Medical Center, PO Box 2040, 3000 CA Rotterdam, the Netherlands.

Corresponding authors
Joanna A.M. Braks, PhD.
Department of Oral & Maxillofacial Surgery, Erasmus Medical Center
Room Ee 230
PO Box 2040
3000 CA Rotterdam, the Netherlands
Phone: +31 10 7038870/ +31 6 25534491
Fax: +31 10 7044685
Email: j.braks@erasmusmc.nl

Jeroen Essers, PhD.
Department of Genetics, Erasmus Medical Center
Room Ee 669
PO Box 2040
3000 CA Rotterdam, the Netherlands
Phone: +31 10 7043604
Fax: +31 10 7044743
Email: j.essers@erasmusmc.nl

Table S1

Primers used for qPCR

mRNA	**Forward (5'-3')**	**Reverse (5'-3')**
VEGF	CGAAACCATGAACTTTCTGCTG	TCCATGAACTTCACCACTTCG
CAIX	AGGGGTCTCTGACTACACCG	GAGGGTGTGGAGCTGCTTAG
CDH1	AATCCCACCACGTACAAGGG	GTGTATACAGCCTCCCACGC
Vim	AGGAGGAAATGGCTCGTCAC	AGAAATCCTGCTCTCCTCGC
Snail	CCAGTGCCTCGACCACTATG	CTGCTGGAAGGTAAACTCTGG
TGFβ1	CGTGGAGGGGAAATTGAGGG	CCGTTGATGTCCACTTGCAG
GAPDH	CACCGTCAAGGCTGAGAACG	GAGGGATCTCGCTCCTGGAAG

Table S2

Increase of tumor bioluminescence signals after xenografting FaDu tumor cells in the floor of the mouth (9-12 mice per group). RT: radiation therapy, HBOT: hyperbaric oxygen therapy.

	Relative tumor bioluminescence (fold increase compared to day 4 ± SEM)					
	day 4	day 7	day 11	day 14	day 18	day 22
control	1	3.12±0.24	10.26±1.17	27.41±3.25	74.05±7.93	
HBOT	1	3.08±0.17	11.86±0.91	31.57±2.44	116.40±15.58	
RT	1	2.60±0.21	2.70±0.37	4.93±0.89	13.72±2.97	26.98±5.73
RT+HBOT	1	2.66±0.15	3.20±0.45	6.04±0.89	14.96±2.44	28.24±5.87
